# *Salmonella enterica* Paratyphi A Infections in Travelers Returning from Cambodia, United States

**DOI:** 10.3201/eid2106.150088

**Published:** 2015-06

**Authors:** Michael C. Judd, Julian E. Grass, Eric D. Mintz, Amelia Bicknese, Barbara E. Mahon

**Affiliations:** Centers for Disease Control and Prevention, Atlanta, Georgia, USA (M.C. Judd, J.E. Grass, E.D. Mintz, A. Bicknese, B.E. Mahon);; Atlanta Research and Education Foundation, Atlanta (M.C. Judd)

**Keywords:** Paratyphoid fever, disease outbreaks, Salmonella enterica, Paratyphi A, Cambodia, drug resistance, United States, travelers, bacteria

**To the Editor:** Health authorities from Cambodia and European Union member states recently described a pronounced increase in *Salmonella enterica* serotype Paratyphi A infections in Cambodia resulting from an ongoing outbreak (*1*,*2*). To further characterize this outbreak, we analyzed 2013–2014 data on Paratyphi A infections associated with travel to Southeast Asia that were reported to the Centers for Disease Control and Prevention (CDC) National Typhoid and Paratyphoid Fever Surveillance (NTPFS) system and the CDC National Antimicrobial Monitoring System (NARMS).

NTPFS began tracking *Salmonella* Paratyphi A infections in 2008. During 2008–2012, ten cases were reported in patients who had traveled to Southeast Asia within 30 days before illness onset; only 1, who also reported travel to Sri Lanka, Nepal, and Nigeria, reported travel to Cambodia. During January 1, 2013–August 22, 2014, however, NTPFS received 19 reports of laboratory-confirmed Paratyphi A infection in travelers returning from Southeast Asia; 13 traveled to Cambodia, and 8 of them reported travel only to Cambodia (Table). Of the 7 patients who traveled only to Cambodia and reported reason for travel, all cited “visiting friends and relatives.” Six (75%) of the 8 patients who traveled only to Cambodia were hospitalized (median duration 7 days, range 2–10 days), and all recovered. Cases occurring in 2014, especially later in the year, might not yet have been reported, so the 2014 data most likely are an underestimate. Although many cases reported to health authorities in Cambodia and the European Union clustered in the Phnom Penh region (*1*,*2*), we lack information about destinations within Cambodia for US patients.

**Table:** Characteristics of patients with *Salmonella enterica* serotype Paratyphi A infection returning to the United States from Southeast Asia, NTPFS, 2013–2014*

Characteristic	Cambodia only, n = 8	Cambodia and other countries in Southeast. Asia, n = 5†	Other countries in Southeast. Asia only, n = 6‡
Travel history§			
Reason for travel, no (%)			
Business	1 (14)	0	3 (60)
Tourism	0	3 (60)	3 (60)
Visiting friends and relatives	7 (100)	3 (60)	0
Missionary work	0	1 (20)	0
Immigration	0	0	1 (17)
Unknown	1 (<1)	0	0
Demographics			
Age, y, median (range)	23 (9–50)	21 (18–59)	39 (25–52)
Female sex, no. (%)	5 (63)	4 (80)	4 (67)
Clinical			
Hospitalized, no. (%)	6 (75)	3 (60)	2 (33)
No. days, median (range)	7 (2–10)	6 (4–7)	3 (1–4)
Recovered, no. (%)	8 (100)	5 (100)	6 (100)
Specimen source, no. (%)			
Blood	7 (88)	4 (80)	4 (67)
Feces	1 (12)	1 (20)	2 (33)

*Cases occurring in 2014, especially later in the year, might not yet have been reported to NTPFS. NTPFS, National Typhoid and Paratyphoid Fever Surveillance system. †In addition to Cambodia, patients also visited Vietnam (2 patients) and Laos (1 patient).  ‡Other countries in Southeast Asia included Indonesia (4 patients) and Thailand (2 patients). §Of patients with known reason for travel. Some patients listed multiple reasons.

Paratyphi A isolates from southern Asia (e.g., India, Pakistan, Bangladesh) often are resistant to the quinolone nalidixic acid or are multidrug resistant (i.e., resistant to ampicillin, chloramphenicol, and trimethoprim/sulfamethoxazole) (*3*), but little is known about antimicrobial drug resistance among Paratyphi A strains from Southeast Asia. However, most outbreak-associated isolates from Cambodia reported by others have been pansusceptible (*1*,*2*). CDC NARMS characterized the antimicrobial susceptibility of isolates from all patients who reported travel only to Cambodia; 7 (87.5%) were pansusceptible, and 1 (12.5%) was resistant to nalidixic acid and had reduced susceptibility to the fluoroquinolone ciprofloxacin. CDC NARMS also tested isolates from all patients who reported travel to Cambodia and other countries in Southeast Asia and from 2 patients who reported travel to other countries in Southeast Asia only; all were pansusceptible.

The Paratyphi A outbreak in Cambodia appears to be large and ongoing. To our knowledge, information about possible sources and risk factors that could help inform prevention activities is not yet available. This outbreak highlights the urgent need for a paratyphoid fever vaccine; although typhoid fever vaccines exist, persons living in and visiting regions of active Paratyphi A transmission have no alternative to relying exclusively on close attention to food and water safety to mitigate risk (*4*). Furthermore, although most isolates from this outbreak appear to have been pansusceptible, antimicrobial drug resistance has emerged quickly among Paratyphi A strains in southern Asia (*5*–*7*). More comprehensive surveillance of antimicrobial resistance among Paratyphi A strains is warranted in Southeast Asia to determine the extent of geographic expansion of resistant strains from southern Asia and to inform treatment options for management of patients. We recommend a systematic outbreak investigation to determine source and routes of transmission.
